# Study on value-based design of healthcare facilities: Based on review of the literature in the USA and Japan

**DOI:** 10.3389/fpubh.2022.883241

**Published:** 2022-09-09

**Authors:** Ying Zhou, Yaonan Sun, Yi Xu, Hao Yuan

**Affiliations:** ^1^Department of Architecture, School of Architecture, Southeast University, Nanjing, China; ^2^Department of Civil Engineering, School of Science, Nanjing University of Science and Technology, Nanjing, China

**Keywords:** value-based design, evidence-based design, architectural planning, healthcare facility, USA, Japan

## Abstract

With limited medical resources, it is of great significance for countries all over the world to explore architectural design methods to enhance the value of medical facilities. Therefore, it is very necessary to carry out an extensive international comparison. In order to grasp the research trend of healthcare facilities in the world, this paper conducts co-occurrence analysis, cluster analysis, and trend analysis of the keywords of 334 English articles and 153 Japanese articles related to healthcare facilities published in important academic journals in the USA and Japan in the past 15 years. We not only compare the similarities and differences in healthcare facility research between the USA and Japan, but also explore the reasons for these differences. It is found that by introducing three kinds of value variables, such as time, space, and behavior, we can not only well explain the difference in the solution of healthcare facilities in the USA and Japan to reduce medical costs but also provide new ideas for architectural design to enhance the value of hospitals. Based on the above analysis, a conceptual framework of value-based design of healthcare facilities that combines the advantages of the USA and Japan is proposed in this paper.

## Introduction

Healthcare systems all over the world are facing the problem of both improving the quality of healthcare and controlling medical costs. Thanks to the booming medical technology, the average human life expectancy has increased rapidly from the 40s before World War II to nearly 80 years at present over the past three-quarters of a century. However, due to the infinite increasing characteristics of healthcare services, medical costs all over the world might increase faster during this period. For example, healthcare costs in the USA reached 16.7% of GDP in 2019 (1). For developing countries, it is also very necessary to effectively control medical costs to improve affordability while improving the quality and accessibility of healthcare systems.

In such context, value-based medicine, which aims to improve the medical outcomes per unit cost, has attracted extensive attention in the world medical community in recent years. For example, lean medicine (2), which aims to reduce unnecessary waste, and value-based competition (3), which focuses on the therapeutic effect of patients, can all be viewed as different aspects of value-based medicine. It could be said that the medical system is so complex, and the meaning of value is so rich that it is necessary to explore value-based healthcare from different perspectives at this stage.

As hospital spending accounts for a large part of the total medical costs (4) and the construction or operation costs of the facilities account for a large part of the hospital spending, it is also very meaningful to improve medical outcomes and reduce costs by improving the design methods of healthcare facilities.

As we all know, healthcare design is very difficult because it not only meets the complex requirements of medical operation processes, medical equipment, and hospital management in the limited space but also coordinates the various possible opposing demands of various stakeholders, such as patients, medical staff, administrators, and even medical payers in the use of space.

In order to achieve such goals, both existing experience-based design approaches and evidence-based design approaches need to be re-examined.

In fact, healthcare design around the world was mainly based on the experience of architects until the 1950s. With the development of medical technology, the size of hospitals is increasing, and the medical functions are becoming more and more complex. In developed countries, such as the USA and Japan, evidence accumulated through scientific research, such as Post-Occupancy Evaluation (POE) survey, has played an increasingly important role in healthcare design, and the currently popular evidence-based design (EBD) approach was formed under such a trend (5). However, EBD is only a local and in-depth research method or design process because most of the existing evidence only reveals the microscopic mechanisms between environmental variables and healthcare outcomes, rather than reflecting the relationship between these mechanisms and the master plan of the facilities.

As healthcare facilities become increasingly complex, the evidence not only accumulates to a very large amount but also often contradicts each other due to differences in research viewpoints, which is very inconvenient for architects to increase the value of healthcare facilities through comprehensive judgment in the early design stage. Therefore, healthcare design needs not only to accumulate new evidence in new usage scenarios but also to select and integrate useful content from a large amount of existing evidence through reasonable value judgment.

Moreover, extensive international comparisons are necessary for the field of healthcare design. For example, although healthcare design in Japan had been heavily influenced by the USA (6), many aspects of Japanese hospitals are still very different from those in the USA, especially in terms of high quality and low price. The reasons behind it need to be further explored through comparative studies.

It cannot be ignored that healthcare design in many populous developing countries is still in the transitional stage from experience-based design to evidence-based design. Due to the relatively weak research foundation, architects from these countries have to learn from a large amount of research results and the evidence accumulated by developed countries, such as the USA and Japan. Only a comprehensive and in-depth understanding of the facts behind the evidence can facilitate a reasonable trade-off in design practice. Otherwise, in order to solve a problem in one aspect, it may bring more problems in other aspects. For example, while healthcare facilities in the USA have advanced medical technology and advanced hospital management experience, their high construction standards may be unaffordable for developing countries. Although healthcare facilities in Japan are inexpensive and accessible, their layout is closely related to their unique nursing system.

All in all, in order to take into account both inherent and universal aspects of international experience, a comparative analysis of research articles related to health facilities published in important academic journals in the USA and Japan from the perspective of a third country is conducted as follows. On this basis, the conceptual framework of value-based design of healthcare facilities of universal significance is then proposed.

## Methods

The following research methods are adopted in this paper to find out the similarities and differences in the field of healthcare design research in detail between the USA and Japan.

First, we selected all health environment and design relevant articles in the past 15 years from a representative English journal and a representative Japanese journal separately in order to grasp the overall research situation and recent hot issues in this field in the USA and Japan.

The English journal is *Health Environments Research & Design Journal* (HERD), which was founded in 2007 (7) by D. Kirk Hamilton who proposed EBD in 2003 (5). The journal is currently the most representative journal in the field of healthcare facility and evidence-based design in the USA. We chose reviews and articles in which research objects are about American medical facilities and the first author's institution is in the USA from HERD for this study and removed articles that do not provide keyword information.

*Architectural Institute of Japan (AIJ) Journals*, which include the *Journal of Architecture Planning, Journal of Structural and Construction Engineering, Journal of Environmental Engineering*, and *AIJ Journal of Technology and Design*, are the top academic journals in the field of architecture in Japan (8). Although the articles published in these journals are of high quality, the value of these articles is not easily recognized by international peers because they are written in Japanese. We selected articles or reviews related to the studies of healthcare facilities in Japan over the past 15 years from AIJ journals.

Second, we used the VOSviewer software to establish a keyword co-occurrence network and make visualization mapping using the analysis method of LinLog and modularity, and then we conducted comparative studies of keyword co-occurrence, cluster, and trend between the articles from HERD and those from AIJ journals.

Third, the concept of value variables is proposed, and the differences between the USA and Japan in care unit design and the reasons behind them are analyzed through value variables, such as the length of hospital stay, bed area, and nursing system.

Finally, we propose a conceptual framework for the value-based design of healthcare facilities and conduct case studies based on it.

## Results

### Preliminary results

**Figure 1:**
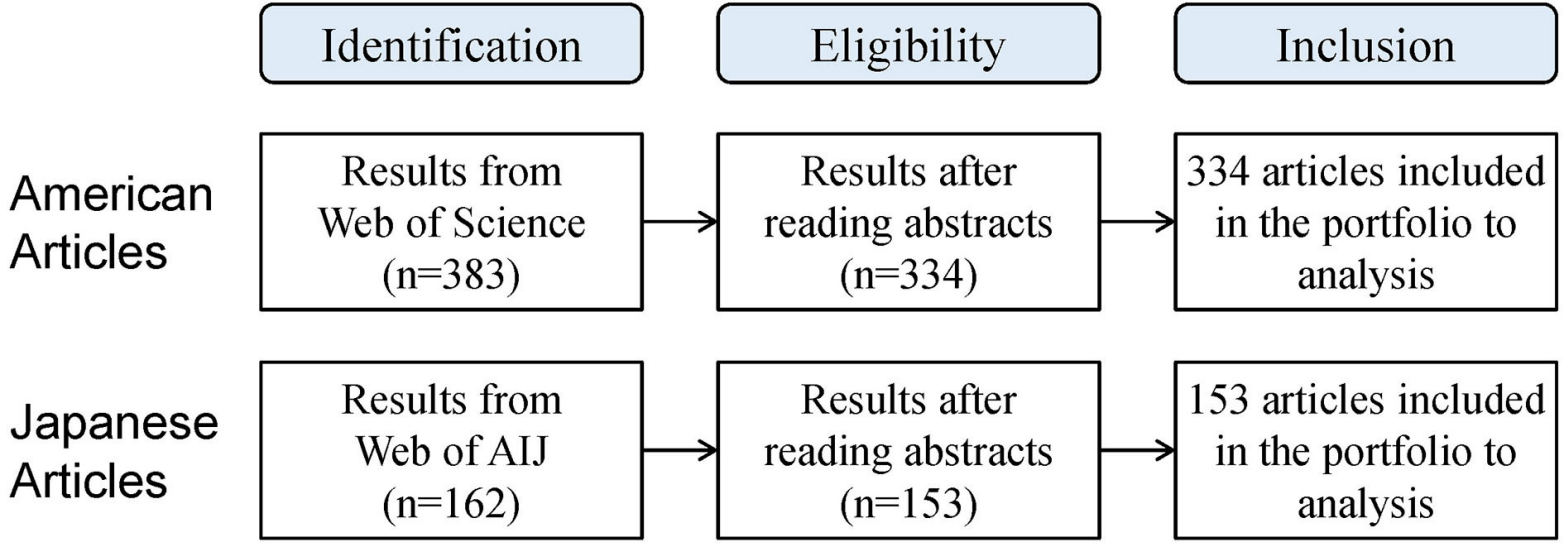
Obtaining the articles for analysis.

Search strategies, topics and publication titles, and results are shown in Table 1. A total of 383 articles in HERD and 162 articles in AIJ Journals are obtained, and 334 eligible HERD articles and 153 eligible AIJ Journals articles were considered for analysis (Figure 1).

**Table 1:** Search strategies and results of the English and Japanese articles.

	**Search strategy**	**Topics and publication titles**	**Results**
English articles	Database: Web of Science	“HERD Health Environments Research Design Journal”	383
	Search in: Publication Titles		
	Type of Article: Review or Article		
	Countries/Regions: the USA		
	Publication Years: FAL 2007-Jan 2022		
Japanese articles	Database: Architectural Institute of Japan	(“byou” or “i” in Chinese characters) and (“Journal of Architecture Planning” or “Journal of Structural and Construction Engineering” or “Journal of Environmental Engineering” or “AIJ Journal of Technology and Design”)	162
	Search in: Topic and Publication Titles		
	Type of Article: Review or Article		
	Publication Years: Jan 2007-Dec 2021		

### Keyword analysis of the articles selected from HERD

**Figure 2:**
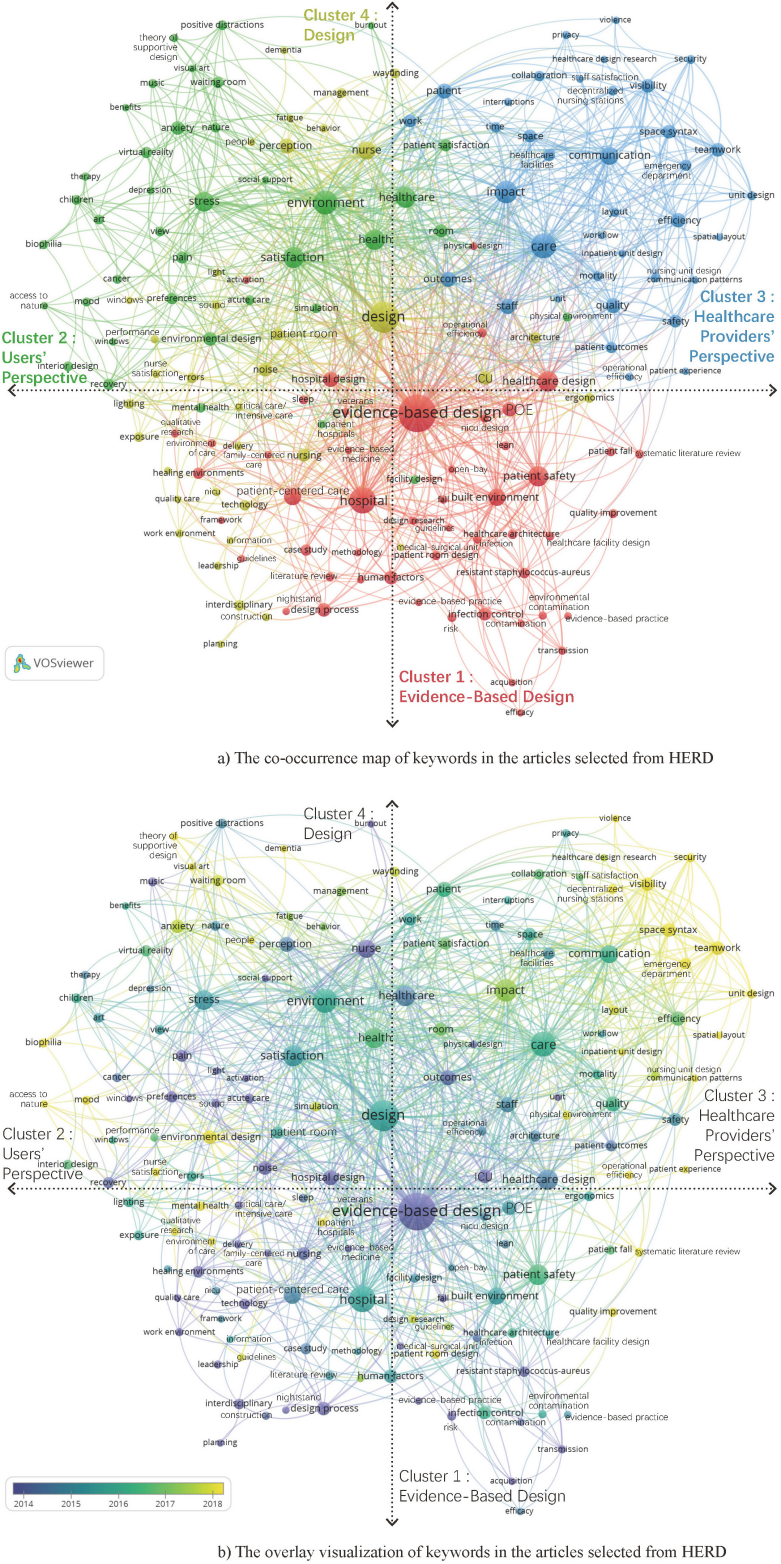
Keyword analysis of the articles selected from HERD. **(A)** The co-occurrence map of keywords in the articles selected from HERD. **(B)** The overlay visualization of keywords in the articles selected from HERD.

Figure 2A shows the co-occurrence map of keywords in the articles selected from HERD. All keywords are divided into four clusters, represented by red, green, yellow, and blue colors, respectively. Table 2 shows that the main keywords in the four clusters are divided into five categories (method, physical space, concerning issues, care, and person).

**Table 2:** Cluster analysis of keywords in the articles selected from HERD.

**Cluster**	**Cluster 1**	**TLS**	**Cluster 2**	**TLS**	**Cluster3**	**TLS**	**Cluster 4**	**TLS**
**Cluster label**	**Evidence-based design**		**Users' perspective**		**Healthcare providers' perspective**		**Design**	
Method	Evidence-based design	488	Environmental design	87	Healthcare design research	25	Design	**347**
	Post-occupancy evaluation	67	Virtual reality	40			Planning	15
	Case study	42	Simulation	19				
Physical space	Hospital, hospital design	322	Environment, physical environment	289	Unit design, nursing unit design, inpatient unit design, unit	162	ICU, NICU	97
	Built environment	121	Room	68	Space, space syntax	155	Patient room	67
	Healing environments	47	Waiting room	41	Layout, spatial layout	70	Architecture	49
	Patient room design	43	Inpatient hospitals	36	Emergency department	60	Work environment	33
	NICU design	26	Interior design	30	Decentralized nursing stations	46	Hospital construction, construction	29
Concerned issues	Patient safety	157	Satisfaction	210	Impact	222	Noise, sound	138
	Infection control, infection healthcare-associated infection	121	Health, mental health	229	Communication, communication patterns	200	Perception	86
	Human factors	73	Stress	186	Efficiency, operational efficiency	137	Lighting, light	74
	Fall, patient fall	66	Anxiety, depression, burnout	145	Teamwork, collaboration	148	Ergonomics	54
	Resistant *Staphylococcus-aureus*	60	Nature, biophilia, access to nature	107	Security, safety	127	Errors	52
	Design process	51	Social support, theory of supportive design	73	Outcomes	109	Wayfinding	46
	Sleep	44	Patient satisfaction	63	Visibility	105	Exposure	41
	Risk	28	Pain	60	Quality	100	Management	39
	Contamination, Environmental Contamination	38	Preferences	56	Work, workflow	90	Fatigue	38
	Transmission	32	Recovery	47	Patient outcomes	68	Technology	37
	Quality improvement	24	View	42	Time	53	Performance	33
	Activation	20	Art, visual art	74	Mortality	40	Behavior	33
	Acquisition	19	Positive distractions	41	Privacy	37	Interdisciplinary	33
	Efficacy	17	Mood	37	Violence	30	Nurse satisfaction	30
	Guidelines	17	Music	34	Staff satisfaction	29	Information	26
	Lean	13	Benefits	32	Patient experience	25	Reliability	23
	Framework	12	Windows	28	Interruptions	14	Leadership	13
Care	Patient-centered care, Family-centered care	142	Healthcare	150	Care	280	Nursing	66
	Intensive care	45	Acute care	37			Critical care/intensive care	40
	Evidence-based medicine	20	Therapy	33			Quality care	27
Person	Veterans	22	Children	45	Patient, staff	226	Nurse	140

Cluster 1 labeled as *Evidence-Based Design* mainly includes the following aspects: (1) patient safety, fall, risk, sleep, and activation; (2) patient-centered care and family-centered care; (3) infection control, contamination, and transmission; (4) human factors, efficacy, and quality improvement, which are important issues for the research-based design of healthcare facilities.

Cluster 2 labeled as *Users' Perspective* mainly includes the following aspects: (1) satisfaction, particularly patient satisfaction; (2) preferences; (3) health, particularly mental health; (4) stress, anxiety, depression, burnout, pain, and mood; (5) nature, art, music, positive distractions, and social support.

Cluster 3 labeled as *Healthcare Providers' Perspective* mainly focuses on the environmental design of efficient and effective care in the nursing unit, emergency department, and so on. The main research topics include the following aspects: (1) impact, efficiency, outcomes, and quality; (2) communication, teamwork, and collaboration; (3) security, safety, visibility, and mortality; (4) work and workflow.

Cluster 4 labeled as *Design* mainly includes the following aspects: (1) noise, sound, lighting, and light; (2) perception, way-finding, and behavior; (3) ergonomics and errors; (4) nurse satisfaction, exposure, and fatigue; (5) management, performance, reliability, etc., which are the important issues for the general design of healthcare facilities.

Table 3 shows the most used keywords in the articles from HERD, with the occurrences, total link strength (TLS), and the clusters to which they belong. It can be found that cluster 1 has the most occurrences and TLS of keywords among the top 25 most frequently occurring keywords, followed by cluster 3. The results also indicate that EBD has a dominant position in the USA healthcare design, and the studies from *Healthcare Providers' Perspective* are still important even in the patient-centered era.

**Table 3:** Top 25 most frequently occurring keywords in the articles selected from HERD.

**Keyword**	**Occurrences**	**TLS**	**Cluster**
Evidence-based design	101	488	Cluster 1
Design	69	347	Cluster 4
Hospital	49	244	Cluster 1
Care	45	280	Cluster 3
Environment	43	266	Cluster 2
Impact	37	222	Cluster 3
Satisfaction	32	210	Cluster 2
Health	31	194	Cluster 2
Healthcare	30	150	Cluster 2
Healthcare design	30	142	Cluster 1
Patient safety	29	157	Cluster 1
Stress	27	186	Cluster 2
Communication	24	162	Cluster 3
Nurse	23	140	Cluster 4
Built environment	21	121	Cluster 1
Patient-centered care	21	118	Cluster 1
Patient	17	127	Cluster 3
Outcomes	17	109	Cluster 3
Quality	16	100	Cluster 3
Staff	16	99	Cluster 3
Perception	16	86	Cluster 4
Hospital design	16	78	Cluster 1
Efficiency	15	109	Cluster 3
Post-occupancy evaluation	15	67	Cluster 1
Teamwork	14	105	Cluster 3

As shown in Figure 2B, the research hotspots represented by yellow color in the USA include the following: (1) using space syntax to study improving visibility and teamwork mainly in emergency departments (9–13); (2) reducing patients' anxiety by having access to nature, music, visual art, etc. mainly in a waiting room (14–17); and (3) nurses' satisfaction (18–21).

### Keyword analysis of the articles selected from AIJ journals

**Figure 3:**
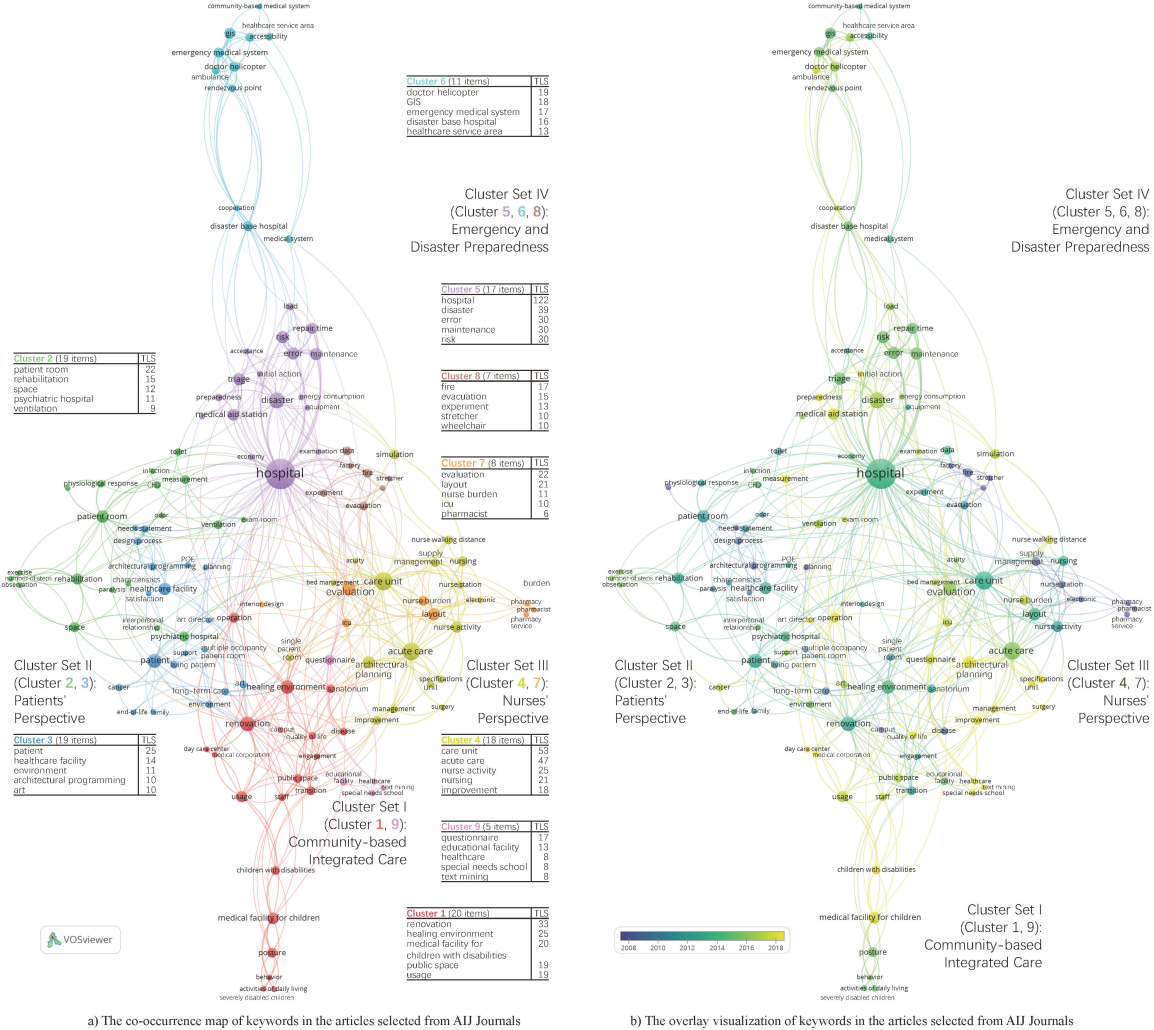
Keywords analysis of the articles selected from AIJ journals. **(A)** The co-occurrence map of keywords in the articles selected from AIJ journals. **(B)** The overlay visualization of keywords in the articles selected from AIJ Journals.

Figure 3A shows the co-occurrence map of keywords in the articles selected from AIJ Journals. All keywords are divided into nine clusters with nine colors. Nine tables in Figure 3A show the top five most frequently occurring keywords of each cluster, respectively. We combined the adjacent or relevant two or three clusters in Figure 3A into one cluster set.

*Cluster Set I* labeled as *Community-based Integrated Care* includes cluster 1 and cluster 9. It mainly focuses on environmental design that contributes to the quality of life for the elderly (22, 23) and persons with disabilities or chronic diseases, especially children with disabilities (24–27) or ALS patients (28, 29), because the community-based integrated care system has been implemented in Japan since 2005.

*Cluster Set II* labeled as *Patients' Perspective* includes cluster 2 and cluster 3. The most frequently occurring keyword in cluster 2 is patient room (30–32) and that in cluster 3 is patient (33–35).

*Cluster Set III* labeled as *Nurses' Perspective* includes cluster 4 and cluster 7. It mainly focuses on the layout of the care unit, ICU, or pharmacy based on nurse activity, nursing, etc. to reduce nurse burden (36–39).

*Cluster Set IV* labeled as *Emergency and Disaster Preparedness* includes clusters 5, 6, and 8. It includes topics like earthquake (40–44), emergency (45–50), and fire (51, 52), etc.

Table 4 shows the most used keywords in the selected articles from AIJ Journals with the occurrences, TLS, clusters, and cluster sets to which they belong. It can be found that *Cluster Set IV* has the highest number of keywords among the top 25 most frequently occurring keywords, followed by *Cluster Set III*. Due to the frequent occurrences of natural disasters in Japan, Japanese researchers have done a lot of research related to *Emergency and Disaster Preparedness*. In addition, studies on optimum design based on analysis of nursing activities to improve efficiency have been paid much attention in Japan?

**Table 4:** Top 25 most frequently occurring keywords in the articles selected from AIJ journals.

**Keyword**	**Occurrences**	**TLS**	**Cluster**	**Cluster set**
Hospital	46	122	Cluster 5	Cluster set IV
Care unit	16	53	Cluster 4	Cluster set III
Acute care	15	47	Cluster 4	Cluster set III
Disaster	13	39	Cluster 5	Cluster set IV
Renovation	11	33	Cluster 1	Cluster set I
Patient	10	25	Cluster 3	Cluster set I
Healing environment	9	25	Cluster 1	Cluster set I
Evaluation	9	22	Cluster 7	Cluster set III
Patient room	8	22	Cluster 2	Cluster set II
Healthcare facility	8	14	Cluster 3	Cluster set II
Layout	8	21	Cluster 7	Cluster set III
Architectural planning	8	10	Cluster 4	Cluster set III
Error	8	30	Cluster 5	Cluster set IV
Maintenance	8	30	Cluster 5	Cluster set IV
Risk	8	30	Cluster 5	Cluster set IV
Triage	8	23	Cluster 5	Cluster set IV
Medical facility for children with disabilities	7	20	Cluster 1	Cluster set I
Medical aid station	7	18	Cluster 5	Cluster set IV
Usage	6	19	Cluster 1	Cluster set I
Rehabilitation	6	15	Cluster 2	Cluster set II
Nurse activity	6	25	Cluster 4	Cluster set III
Repair time	6	24	Cluster 5	Cluster set IV
GIS	6	18	Cluster 6	Cluster set IV
Nursing	5	21	Cluster 4	Cluster set III
Doctor helicopter	5	19	Cluster 6	Cluster set IV

As shown in Figure 3B, the research hotspots since 2018 in Japan include the following: (1) environmental design that contributes to the quality of life for children with disabilities in *Cluster Set I*; (2) the way to promote the widespread use of healing arts in the hospital (53) in *Cluster Set II*; (3) reducing nurse burden and walking distance through the reasonable layout of ICU and acute care unit in *Cluster set III*; and (4) optimizing the design of medical aid stations and improving the initial action system of disaster base hospital by careful preparedness in *Cluster Set IV*.

### Comparative study between the USA and Japan

**Figure 4:**
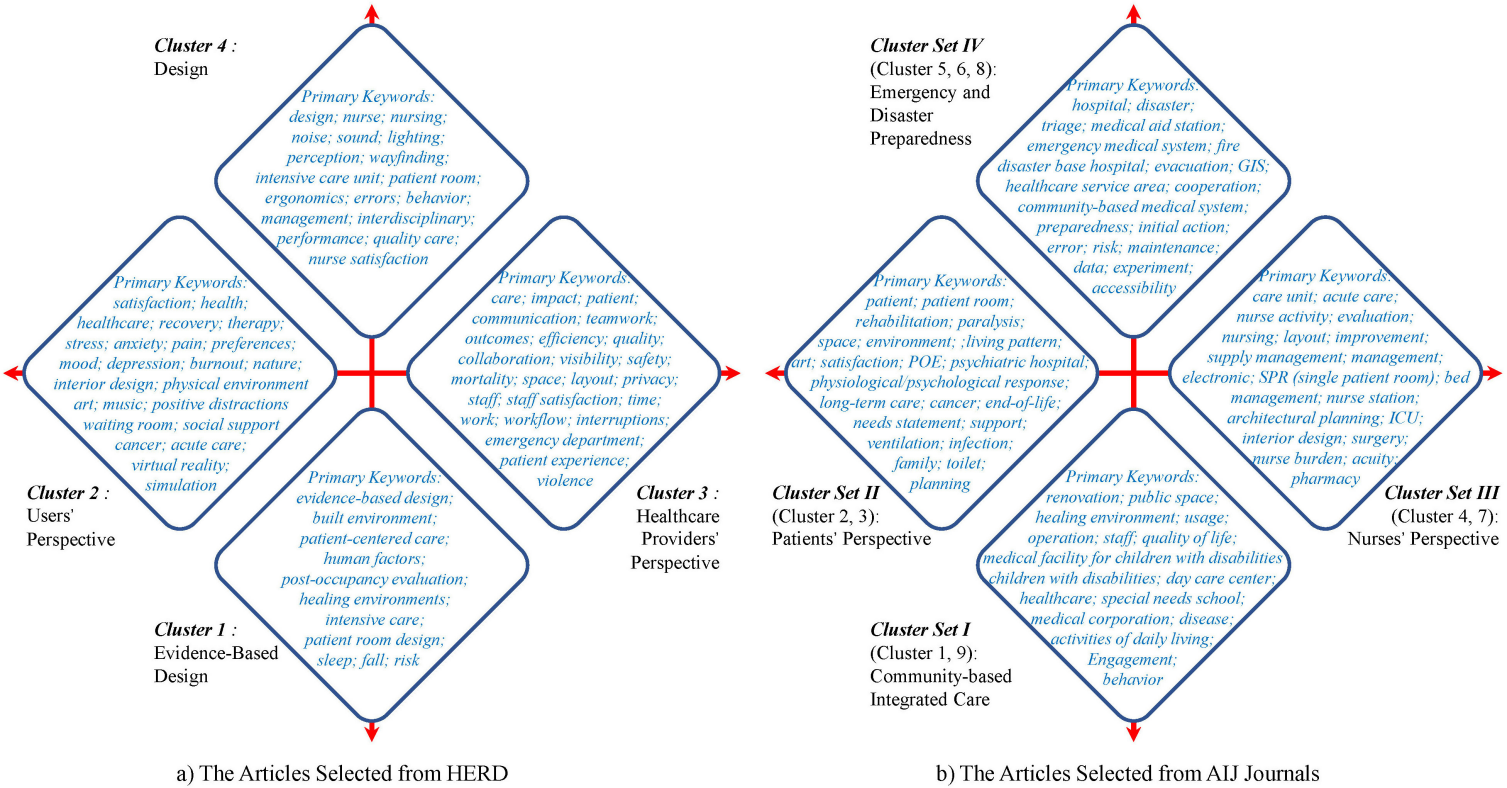
Comparative study on characteristics of four clusters or cluster sets between the USA and Japan. **(A)** The articles selected from HERD. **(B)** The Articles Selected from AIJ journals.

The 4 cluster/cluster set labels and the most frequently occurring keywords in the articles selected from HERD and AIJ Journals are shown in Figures 4A,B, respectively. It can be found that there are both similarities and differences in research related to healthcare facilities between the USA and Japan.

First, the two sides of the horizontal axis in Figure 4A represent the *Users' Perspective* and *Healthcare Providers' Perspective*, respectively, and the two sides of the horizontal axis in Figure 4B represent the *Patients' Perspective* and *Nurses' Perspective*, respectively. The research objects of the USA and Japan on the horizontal axis are similar, indicating that both users and healthcare providers are key research topics in healthcare facilities in different countries.

Second, the longitudinal axis in Figure 4A is quite different from that in Figure 4B. The upper and lower sides of the vertical axis in Figure 4A represent *Design* and *EBD*, respectively. The *Design* cluster focuses on general design issues, while *the EBD* cluster focuses on design issues that require in-depth study. The upper and lower sides of the vertical axis in Figure 4B represent *Emergency and Disaster Preparedness* and *Community-based Integrated Care*, respectively, because Japan is prone to many disasters and has an aging population.

The *care unit design* is taken as an example to further discuss the similarities and differences in healthcare design research between the USA and Japan, which is described in detail in the following section.

#### Care unit design

##### HERD

###### Length of stay

As one of the main means of reducing medical costs, the USA has paid great attention to research that can shorten the length of stay. The results published in HERD articles suggest that factors that can shorten the length of stay include sunlight (54), views of nature (54), patient-centered model of care (55), acute care unit for the elderly (56), adaptive healing room (57), expedient bariatric beds (58), therapeutic design (59), and proximal to the ward entrance (60, 61). Concentration or decentralization of nurse stations has no significant effect on the average length of stay (62, 63).

###### Design of patient rooms

According to the Facility Guidelines 2006, the minimum bed area, the recommended bed area, and the bed area including family space are 120 ft^2^ (11.2 m^2^), 160 ft^2^ (14.9 m^2^), and 250 ft^2^ (23.2 m^2^), respectively (64). Research shows that accommodating patients in single-occupancy patient rooms (SPRs) instead of traditional MPRs (multi-bed patient rooms) can improve numerous healthcare outcomes, such as sleep quality, privacy, communication between patients and staff, satisfaction of patients and families, and reduce hospital-acquired infection rates, stress level, patient transfer, and length of stay (65).

**Figure 5:**
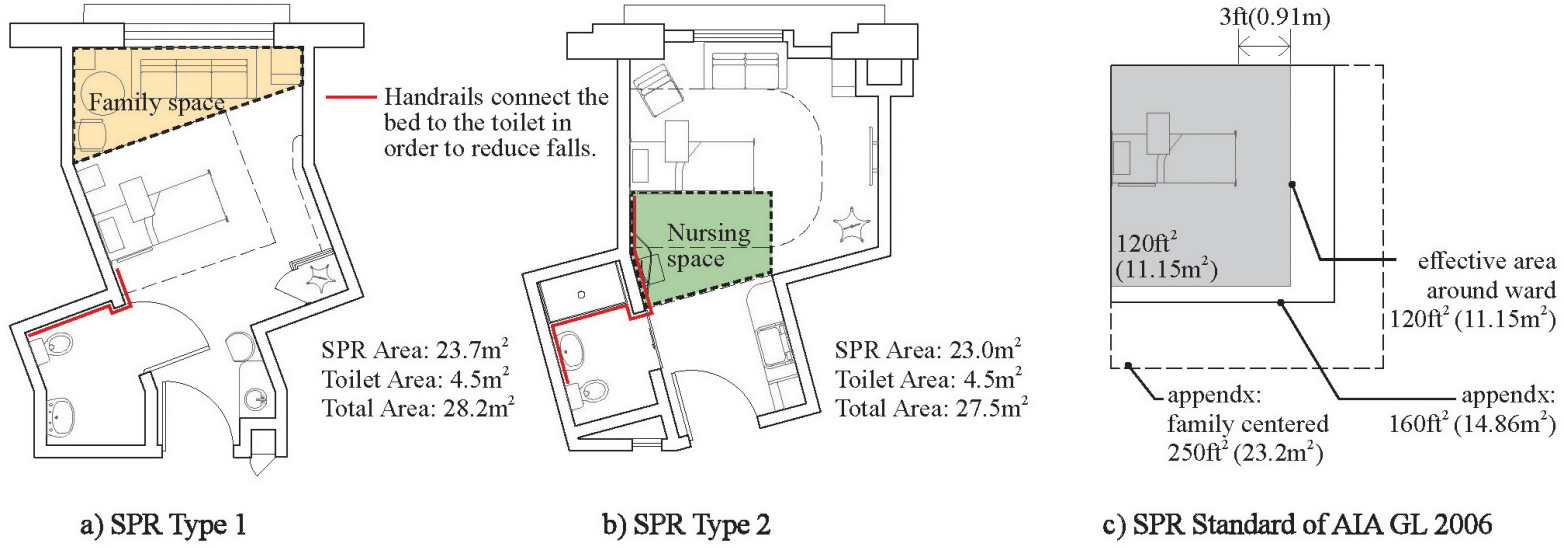
Typical SPRs in the USA. **(A)** SPR Type 1. **(B)** SPR Type 2. **(C)** SPR Standard of AIA GL 2006.

The SPRs in the USA have been studied in depth on preventing patients from falling (66), family- and staff-supported spaces, etc. As shown in Figure 5, the typical US single PR has the following advantages: (1) short distance and few turns between beds and toilets for fall prevention; (2) comfortable family-supported space; and (3) staff-supported space for bedside care at ease (67). In addition, the design of the special patient rooms for the elderly (68) or terminal patients (69) has also been successful.

However, the construction standard of SPRs in the USA, especially the acuity-adaptable SPRs (70, 71) developed to reduce intra-hospital patient transfer, is too high to control medical costs.

###### Nurses' stress and satisfaction

Healthcare providers, especially nurses, experience a high level of work stress (54). Studies that examine how the physical environment contributes to reducing staff stress are very significant. The relationships between nurses' stress and physical elements, such as exterior views, daylighting, lighting, color, spatial color patterning, and noise control, were widely discussed (20, 54, 72–75). The thoughtful design of the lighting environment can improve nurses' satisfaction (21, 76). The restorative quality of break areas may significantly improve nurses' satisfaction and stress reduction, potentially leading to improved care for the patients they serve (77). The layout design that can reduce nurse walking or enhance teamwork in care units can improve nurses' satisfaction (18, 78).

Single-bed ICU design was associated with higher levels of stress for ICU nurses (79), and the single-family room model in the NICU can cause staff members to feel isolated from one another and reduce their ability to respond quickly in a crisis situation (80).

#### AIJ journals

##### Type of beds and care units

General hospital beds in Japan are divided into four categories: intensive care beds, acute care beds, convalescent care beds, and chronic care beds. The average length of stay of the first three types of beds reached 16 days, which is much higher than that of European and American countries. With the aging of the population, Japan has been reducing the proportion of acute care beds and increasing the proportion of convalescent care beds while reducing the total number of hospital beds. According to Japan's regional medical care vision, acute care beds will fall from 48% in 2015 to 34% in 2025, while convalescence care beds will rise from 10 to 31% (81).

There are four articles on ICU (37), 17 articles on acute care unit (39), and six articles on convalescent care unit (82) in AIJ articles, respectively. These articles mainly aim at how to offer more suitable care and built environment for the treatment of intensive, acute, and convalescent care patients in order to create a coordinated, seamless medical system.

##### Design of patient room and care unit

**Figure 6:**
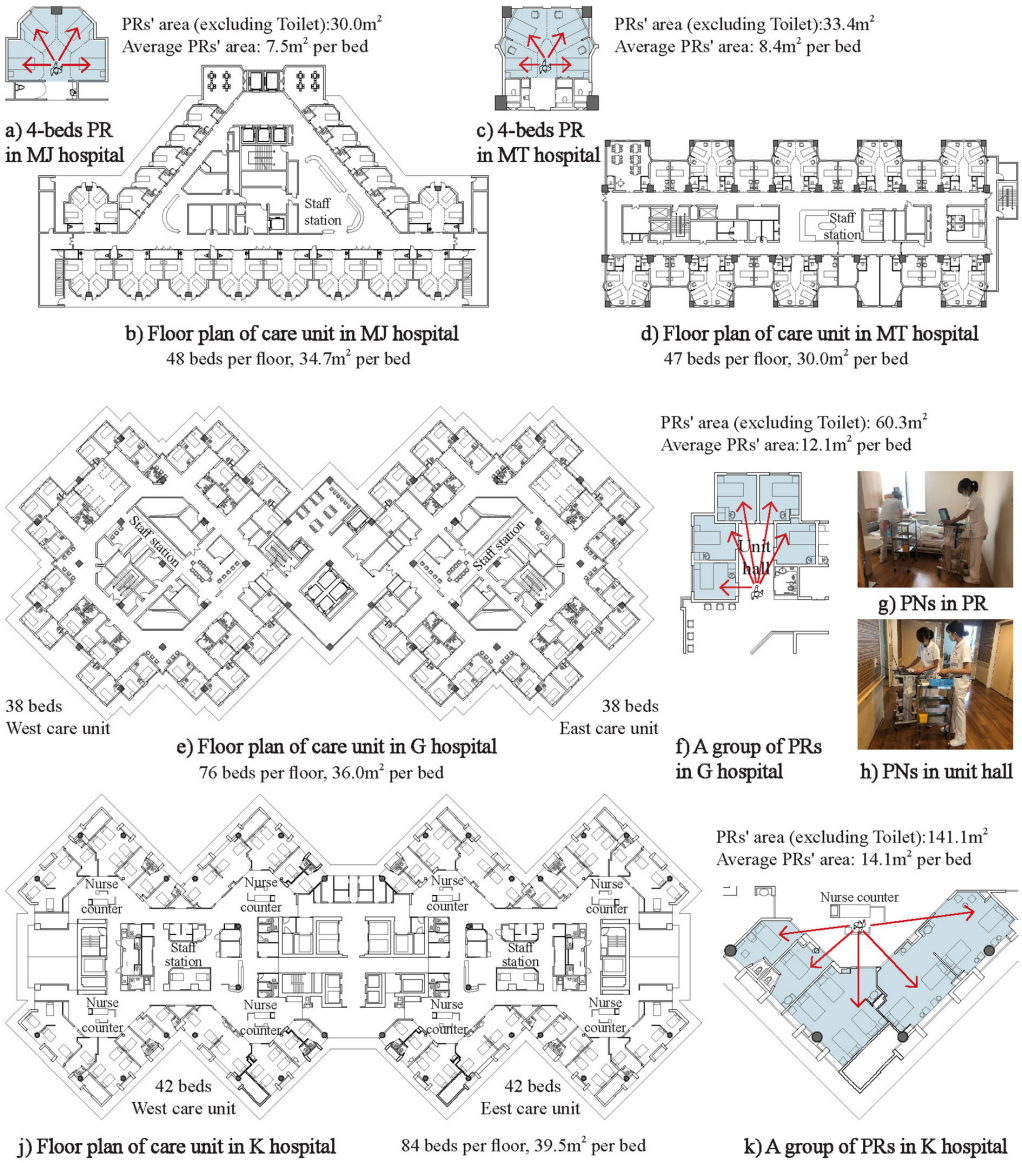
Typical patient rooms in Japan. **(A)** 4-beds PR in MJ hospitalin hospital PRs' area (excluding Toilet):30.0m^2^, Average PRs' area: 7.5m^2^ per bed. **(B)** Floor plan of care unit in MJ hospital 48 beds per floor, 34.7m^2^ per bed. **(C)** 4-beds PR in MT hospital PRs' area (excluding Toilet):33.4m^2^ Average PRs' area: 8.4m^2^ per bed. **(D)** Floor plan of care unit in MT hospital 47 beds per floor, 30.0m^2^ per bed. **(E)** Floor plan of care unit in G hospital 38 beds West care unit 38 beds East care unit 76 beds per floor, 36.0m^2^ per bed. **(F)** A group of PRs in G hospital PRs' area (excluding Toilet): 60.3m^2^ Average PRs' area:12.1m^2^ per bed **(G)** PNs in PR. **(H)** PNs in unit hall. **(J)** Floor plan of care unit in K hospital.84 beds per floor, 39.5m^2^ per bed 42 beds West care unit 42 beds East care unit **(K)** A group of PRs in K hospital PRs' area (excluding Toilet):141.1m^2^ Average PRs' area: 14.1m^2^ per bed.

Both SPRs and MPRs have their own advantages and disadvantages. Despite the disadvantages of poor patient privacy, the MPRs are cheap, short in nurse walking, and convenient for nursing observation and nursing collaboration. Unlike all SPRs adopted in the US hospitals, MPRs still account for a considerable proportion in Japanese hospitals. Especially in recent years, Japan's four-bed PRs have shown many new characteristics by absorbing the traditional advantages of SPRs. As shown in Figures 6A,C, with an individual territory and window for each patient, it is not only convenient for nursing observation and patient transfer but also avoids sight interference between patients effectively (83). At the same time, the average bed area, length of the corridor, and nurse walking do not increase significantly.

In Japan, critically ill patients who require close observation are usually placed near the nurse's station and then transferred when the patient's condition improves. In order to reduce the nurses' burden due to patient transfer, some Japanese hospitals have adopted SPRs with lower standards and close to the nurse station (Figure 6E) (84, 85), which can take into account the privacy of patients and the convenience of staff.

##### Nurse activities

Studies on nurse activities have always been concerned in Japan (86). Especially in recent years, due to the wide application of Hospital Information System (HIS) and Supply, Processing, and Distribution (SPD) System, the improvement in the nursing system and the increase in the number of SPRs have had a great impact on nurse walking. The main related research results are as follows.

Only the decentralized nurse stations equipped with HIS (87, 88) and the corresponding medical supplies system (89) can play roles in shortening the walking distance of nurses. The walking distances of two nurses are used as an evaluation index of the Partnership Nursing System (PNS) to discuss the level of equality as an equal nursing partner (84).

Unlike the traditional follow-up survey, many new technologies have been applied in the study of nurse activities in recent years. For example, ultrasound positioning technology has been used to collect data on nursing activities consecutively for 1 week (90), and BIM technology is used to optimize the bed position of inpatients and simulate the walking distance of nurses (84, 91).

#### Summary

Overall, the USA studies of care units focus on the healthcare environment that could shorten the length of stay and improve patient and staff satisfaction, while Japan focuses on the spatial layout to improve the efficiency and effectiveness of nursing care. Taking patient satisfaction as an example, there have been 90 related articles in HERD since 2007, accounting for 26.9% of the total, while there are only two related articles in AIJ Journals, accounting for 1.3% of the total.

Research goals are also significantly different between the USA and Japan. In the case of studies related to the physical environment, such as indoor environment or view outside the window, most Japanese studies just stay at the level of reducing patient or staff stress and anxiety, while American studies will even achieve the level of performance, such as healing effects or length of stay (64).

As for research methods, EBD occupies a central place in the USA, while Japanese architectural planning mainly refers to POE analysis or computer simulation based on field surveys.

## Discussion

### Cost-benefit analysis

As shown in Table 5, from the perspective of medical costs, the number of doctors (92) and nurses (93) per 1,000 inhabitants in the USA and Japan are almost the same, while the average length of stay in acute care beds (94) and the number of acute care beds per 1,000 inhabitants (95) in Japan are much higher than those in the USA. On the other hand, from the perspective of medical benefits, Japan's medical costs as a proportion of total health spending in GDP (1) are much lower than those in the USA, but the average life expectancy (96) is much higher than that of the USA. It is not so difficult to find that the Japanese healthcare system is more cost-effective than that of the USA just by doing a simple cost-benefit calculation. Of course, the reasons behind the above phenomenon are very complex, and the following analyses of healthcare facilities are made by introducing the concept of value variables.

**Table 5:** OECD data (2019).

**Country**	**Average length of stay in acute care beds**	**Number of acute care beds per 1,000 inhabitants**	**Number of doctors**		**Number of nurses**		**Proportion of total health spending in GDP**	**Life expectancy at 65 (women /men)**
			**Per 100 beds**	**Per 1,000 inhabitants**	**Per 100 beds**	**Per 1,000 inhabitants**		
Japan	16.0	12.8	19.2	2.6	90.6	11.9	11.0	24.6/19.8
Germany	7.4	7.9	55.5	4.4	176.2	11.8	11.7	21.4/18.8
France	5.4	5.8	54.3	3.4	189.6	11.1	11.1	23.9/19.8
United Kingdom	6.2	2.5	120.1	3.0	334.2	8.2	9.9	21.1/18.3
USA	5.4	2.8	92.2	2.6	420.2	12.0	16.7	20.8/18.2

### Value variables

As mentioned above, the research topics and solutions for the healthcare environment with limited medical resources are quite different in the USA and Japan. Through further comparative study between the USA and Japan, we found that time variables such as length of stay, space variables such as bed area and the number of care units per floor, and behavior variables such as nurse activities and nurse system play key roles in care unit design. In this paper, these variables are defined as value variables (Table 6).

**Table 6:** Summary of the relationships between value variables and outcomes.

**Value variables**			**Outcomes**	**Medical accidents**	**Nurse stress**	**Nursing observation**	**Nurse collaboration**	**Interdisciplinary communication**	**Bed management**	**Construction & operating costs**
			**Country**							
Time	Length of stay (day)	5.4	USA	More	High					
		16	Japan	Less	Low					
Space	Bed area (m^2^)	23.2 and above SPRs	USA			Difficult	Difficult		Easy	High
		7.5~14.1 MPRs + SPRs	Japan			Easy	Easy		Difficult	Low
	Number of care units per floor	1~2	USA, Japan	More				Difficult	Difficult	
		3~4		Less				Easy	Easy	
Activity	Nursing system (NS)	Primary NS	USA, Japan		High		Difficult			
		Partnership NS	Japan		Medium		Easy			
		Team NS	USA, Japan		Low		Difficult			

First, we discuss the length of hospital stay. The average length of hospital stay in acute beds has fallen from 8.3 days in 1969 to 5.4 days in 2019 in the USA and from 34.4 days in 1994 to 16.0 days in 2019 in Japan in order to cut medical costs (94). We found that when the length of hospital stay falls below a certain threshold, there will be significant changes in the type of patient rooms, the layout of care units, the nursing system, and so on. When the average length of hospital stay is long enough, progressive patient care is considered as an efficient way (97), and it is better to divide the care units into different types, such as intensive care unit, step-down care unit, and acute care unit in order to offer more suitable care and environment for the corresponding type of patients. On the contrary, when the average length of hospital stay is quite short and inpatients become relatively serious, not only does the classification of care units have little significance, but it is also advisable to use all SPRs in care units (66). However, if the average length of hospital stay falls too short, it may lead to a significant increase in the length of the nurse walking, the difficulties of nursing observation, and nurse stress, which will increase the construction and operating costs of the hospital.

Second, the minimum bed area of acute care units is 6.4 m^2^/bed in Japan, which is only half of the AIA standard (64). In addition, the average number of beds in acute care units in Figure 6 is usually about 42 beds, and the average area of the acute care units is about 1,520 m^2^. The care unit in Japan is more compact compared with that in the USA, which is conducive to shortening the nurse walking and improving the effect of nursing observation and nurse communication. It should be noted that although the bed area in Japan is not large, it does not seem to have any adverse effects on the quality of care or safety (64). Therefore, it is necessary to re-examine the suitable size of the bed area.

Japan has advantages in the nursing system. For example, PNS is a popular nursing system with a pair of two nurses working together to provide care to patients. It can help nurses learn from each other and reduce stress and errors, thereby improving safety and quality. However, PNS needs to be bound closely to the care unit planning. It is necessary to increase the bed area (Figure 6G) and outdoor staff-supported space (Figures 6F,H,K) appropriately because there are always two nurses working together (84, 85, 98).

Using value variables can help us understand or solve problems in healthcare design from three dimensions of time, space, and behavior rather than just one of them. Otherwise, it is easy to create more new problems in order to solve some problems, which will bring unnecessary complexity. For example, cutting medical costs simply by shortening the length of hospital stay may lead to the all-use of SPRs, which may eventually backfire. In addition to the indoor environment, the nursing system can also be taken into account to reduce nurse stress, which may require only small changes in the care unit planning.

### Value variables and environment design variables

The intent of EBD is to base the design decisions on credible studies of healthcare facilities. However, environment design variables used in EBD, such as windows, SPRs, and comfortable waiting rooms, are useful during the phase of design development or interior design, but not during the schematic design phase.

In the schematic design phase, the items like building shapes or traffics, which are needed to be determined by architects, are more closely related to the value variables, such as length of stay, number of care units per floor, and the nursing system.

### Conceptual framework of value-based design

**Figure 7:**
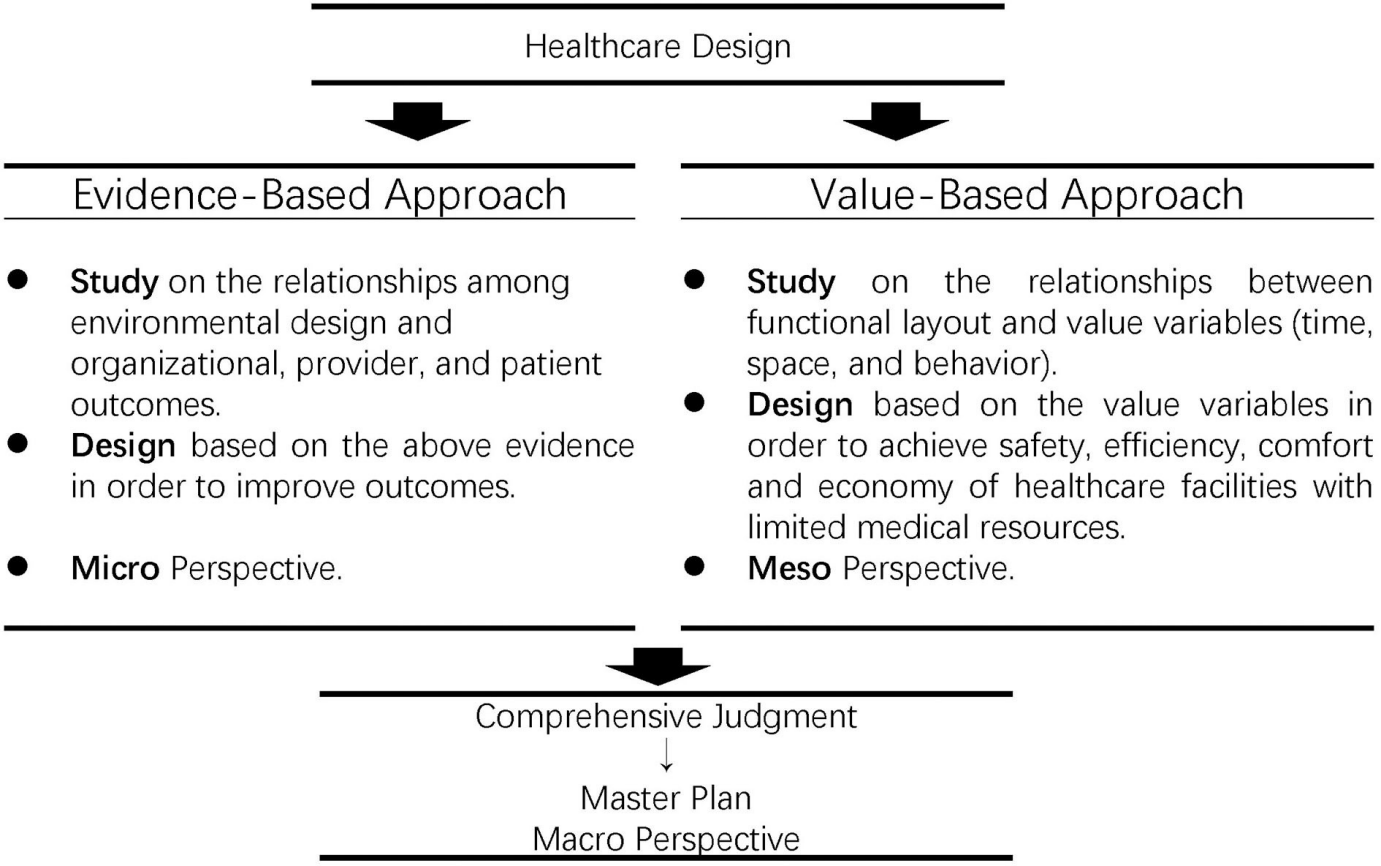
Conceptual framework of value-based design.

The effect of healthcare design depends largely on the architect's comprehensive judgment from a macro-perspective, while the healthcare facility research represented by EBD mainly explores the relationship between environmental variables and specific outcomes from the micro-level. As the importance of evidence is different in different scenarios, evidence choice according to the actual situation is important for healthcare design. In order to cover the gap between healthcare design and research, we propose a framework for the value-based design of healthcare facilities from the meso-level on the basis of EBD and Japanese architectural planning (Figure 7).

In this paper, value-based design is defined as the master planning and evidence choice through value variables (time, space, and behavior) to balance safety, efficiency, comfort, and economy in healthcare facilities with limited medical resources.

**Figure 8:**
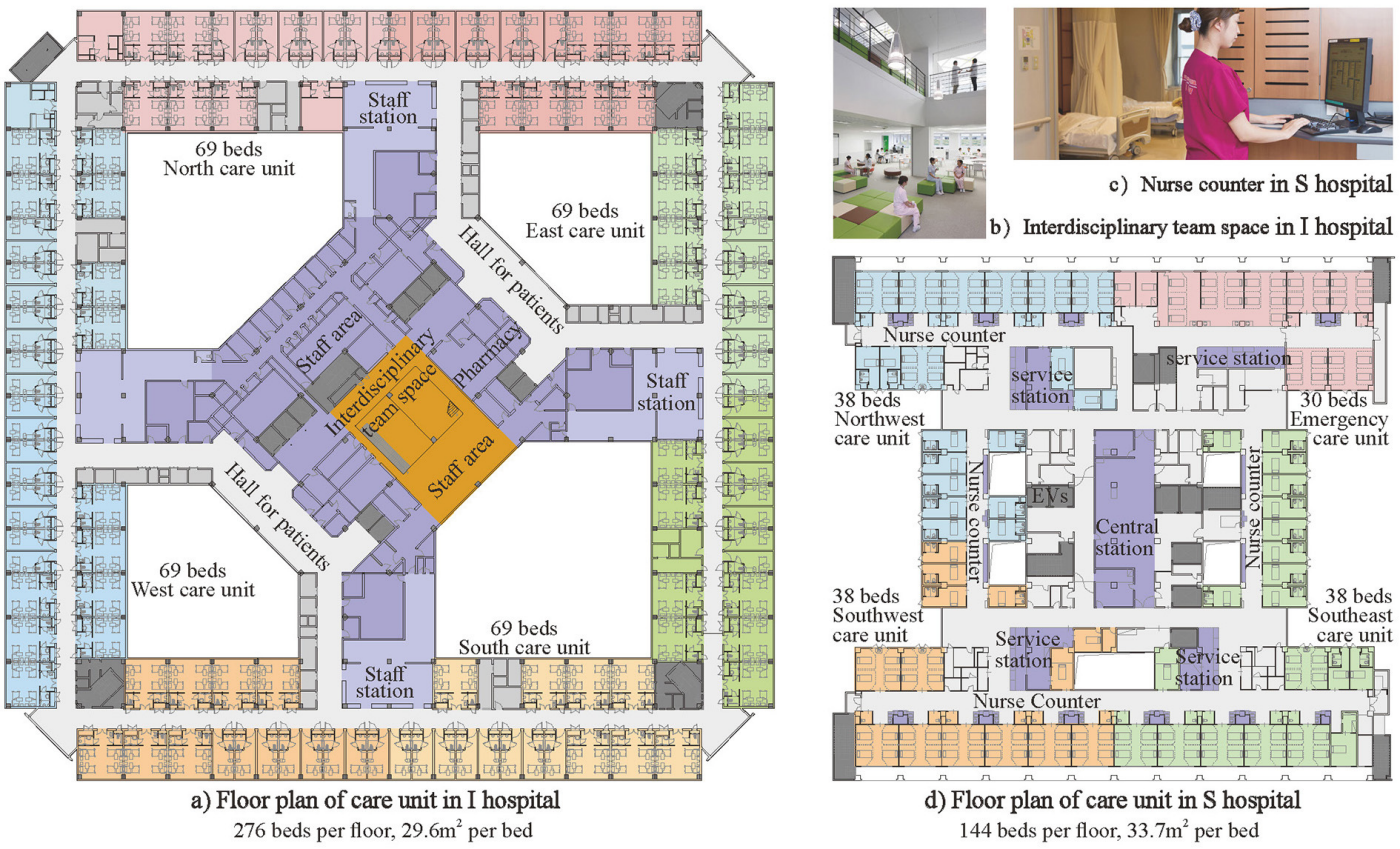
Four care units on one floor. **(A)** Floor plan of care unit in I hospital 276 beds per floor, 29.6m^2^ per bed. **(B)** Interdisciplinary team space in I hospital. **(C)** Nurse counter in S hospital. **(D)** Floor plan of care unit in S hospital 144 beds per floor, 33.7m^2^ per bed.

As shown in Figure 8A, instead of the usual two care units per floor, there are four care units per floor in Y hospital with 655 beds in total. According to this spatial variable, an open public space for an interdisciplinary team Figure 8B is designed in the center of the floor to facilitate formal or informal communication among different specialized professionals involved in healthcare with the overarching goal of improving the safety and quality of patient care.

As shown in Figure 8D, Hospital S is a cardiology hospital with four care units per floor. Because heart disease is a high mortality disease and some heart attacks strike suddenly, the value variable of emergency time needs to be comprehensively considered together with the space and behavior value variables in a hospital design. Therefore, in terms of space, four nursing units are combined into one management unit to flexibly adjust the number of beds in each nursing unit. In addition, in terms of behavior, nursing activities are divided into three categories, such as office work, drug preparation, and bedside nursing. One central station, four service stations, and multiple nurse counters (Figure 8C) are designed in the according location for efficient three levels of care.

## Conclusion

1. The comparative study of the keywords of HERD and AIJ articles can help researchers and designers to grasp the research system of health facilities in the USA and Japan as a whole and to clarify the position of one research topic in the system and the relationship with other research topics.
2. The USA and Japan have their own strengths in the study of healthcare facilities. EBD occupies a central place in the USA, focusing on revealing the micro-mechanisms between environmental variables and healthcare outcomes. The architectural planning approach adopted in Japan focuses on the relationship between hospital layout and improving care processes, increasing medical efficiency, and reducing health care costs.
3. The differences reflected in the solutions of American and Japanese hospitals in controlling medical costs can be well explained by introducing three kinds of meso-level value variables of time, space, and behavior. Value-based design that combines the advantages of healthcare design in the USA and Japan can largely bridge the gap between macro master plans and micro evidence, which can help architects select, integrate, and absorb the most appropriate research results in healthcare design.
4. Through the coordination between different types of value variables, the concepts and methods of value design help to achieve the balance of safety, efficiency, comfort, and economy of healthcare facilities under limited medical resources.
5. The differences in the value of value variables in different countries reflect the different cultural concepts and value orientations behind them, and the understanding of this point can help architects in developing countries to learn from the experience of developed countries.
6. The limitation of this paper is that it only discusses the value variables of the inpatient department, and further study is required for the outpatient department and the medical technology department. The authors also hope to take this study as an opportunity to trigger more discussions in the academic community.

## Author contributions

YZ and YS conceived and designed the analysis, collected the data, performed the analysis, and wrote the paper. YX contributed to figure preparation. HY collected some data. All authors contributed to the article and approved the submitted version.

## Funding

This research was supported by the National Natural Science Foundation of China (Grant No. 51978143) and the Healthy Building Industry Technology Strategic Alliance.

## Conflict of interest

The authors declare that the research was conducted in the absence of any commercial or financial relationships that could be construed as a potential conflict of interest.

## Publisher's note

All claims expressed in this article are solely those of the authors and do not necessarily represent those of their affiliated organizations, or those of the publisher, the editors and the reviewers. Any product that may be evaluated in this article, or claim that may be made by its manufacturer, is not guaranteed or endorsed by the publisher.
